# Legal Counsel, Moral Expectations, and Youth with Intellectual and Developmental Disabilities: Economies of Worth in Youth Courts

**DOI:** 10.1177/09646639251349395

**Published:** 2025-06-13

**Authors:** Dale Spencer, Nathan Innocente, Daniella Bendo

**Affiliations:** 6339 Carleton University, Canada; 71637 University of Toronto, Canada; 113609 University of Western Ontario, Canada

**Keywords:** youth, intellectual and developmental disabilities, situated moral judgment, orders of worth, legal counsel, disability justification

## Abstract

The Youth Criminal Justice Act (YCJA) is the law that governs the Canadian youth justice system and applies to young people between the ages of twelve and seventeen. The YCJA's Declaration of Principle broadly states that measures taken against young people should consider their “special requirements.” Such vague provisions have led young people with mental health issues and intellectual and developmental disabilities to not receive much-needed and legally required accommodations. Based on interviews with 38 legal professionals, we analyze their interpretations of the failure of the youth justice system to accommodate youth with intellectual and developmental disabilities in conflict with the law. We draw from Boltanski and Thevenot’s model of situated moral judgment to understand crown attorney and defense counsel's reflections on, and criticisms of, their work environments in relation to the positioning of young people with intellectual and developmental disabilities.

## Introduction

The Youth Criminal Justice Act in Canada provides that the court or prosecutor can order the medical, psychiatric, or psychological assessment of a young person if “the court has reasonable grounds to believe that the young person may be suffering from a physical or mental illness or disorder, a psychological disorder, an emotional disturbance, a learning disability or a mental disability” (YCJA, 2004: section 34). In this study we discovered that such “reasonable grounds” offers little in the way of guidance of such grounds, fails to consider the economic constraints to providing such assessments, and as such, legal professionals working in the courts largely *suspect,* but do not know, that justice-involved young people in the court have a disability of some sort. We focus on youth with intellectual and developmental disabilities, defined as cognitive and adaptive functioning impairments that involve challenges with everyday living skills. Legal professionals are not unaware of these challenges and recognize the problems associated with working with youth in conflict with the law, and in particular, with young people who have intellectual and developmental disabilities (herein IDD).

In this article, we draw from Boltanski and Thevenot's (2000) model of situated moral judgment to understand crown attorney and defense counsel's reflections on and criticisms of their work environments in relation to the positioning of young people with IDD that are in conflict with the law. We analyze the language used to formulate their criticisms of legal situations presented throughout the justice system process, the material conditions under which they work, and their conceptions of justice in the absence of formal guidelines regarding how to accommodate the needs of and ensure the protection of the rights of young people with IDD. We principally demonstrate that legal professionals engage in three orders of worth that correspond to market, civic, and disability justifications that are the basis of evaluation and criticisms of the youth criminal justice system and its treatment of young people with IDD.

This article is structured in three main sections. First, we outline the Youth Criminal Justice Act in Canada in relation to youth with disabilities. In relation to the extant literature on the contemporary character of legal work, we introduce Boltanski and Thevenot's sociology of situated judgment as an approach to analyze legal counsel's interactions with young people with IDD that are in conflict with the law. This is followed by the methods of this study. The third section provides the results of the research, focusing on how legal professionals conceptualize justice in relation to young people with IDD in the absence of formal guidelines regarding justice outcomes for these young people.

## Legal Work, Moral Expectations, and Young People

In response to some of the criticisms and weaknesses of earlier youth legislations in Canada, legislators passed the Youth Criminal Justice Act (YCJA) (2003–present). The YCJA is the law that governs the Canadian youth justice system, and it applies to young people between the ages of twelve and seventeen. Similar to youth offender-specific legislation in the United States and the United Kingdom, the YCJA provides a legal framework for responding to breaches of criminal law committed by youth before they reach the age of eighteen. The YCJA is designed to (i) hold young people accountable through measures that are proportionate to the seriousness of the offense and the degree of responsibility of the young person; (ii) promote rehabilitation and reintegration; (iii) address underlying causes of justice involvement and, “respond to the needs of young persons with special requirements” (s. 3(c) Declaration of Principle).

Research illustrates that young people in Canada experience inequalities within the criminal justice system, barriers to their rights, and access to justice across various stages in the criminal process—arrest, diversion, bail, trial, and sentencing (McMurtry and Curling, 2007). Justice professionals report issues that include a vague category of “mental disorder” provisions under criminal law, a lack of effective training about IDD, and limited appropriate resources. These systemic barriers create challenges for interacting with youth with IDD and tailoring effective responses for them (Marinos et al., 2008).

Similar to research in the United States (Olley and Cox, 2021; Rogal, 2017) and the United Kingdom (Clare and Gudjonsson, 1991), young people with IDD are not easily identified as having challenges in comprehension or decision-making by those working in the justice system because they are overly compliant and suggestible, and they often seek to appear competent because of the stigma attached to disability (Marinos and Whittingham, 2020). Without appropriate responses to youth with IDD, these individuals are unable to access justice and participate meaningfully in their case as required by the *YCJA*, being further marginalized by a justice system meant to support them, or most seriously, being wrongfully convicted (Marinos, 2017; Marinos et al., 2008). These issues are important because, despite the impact that they may have on young people directly, these are also issues that many justice professionals navigate in their work with youth and within their institutions.

In the Canadian youth justice context, the term disability is oftentimes conflated with other terms such as mental or intellectual disability or disorder. This is unsurprising considering the YCJA's Declaration of Principle broadly states that measures taken against youth should consider their “special requirements” (Section 3(c)(iv)) but does not provide a specific definition for conceptualizing disabilit(ies). Understanding disabilities and youth justice in the Canadian context is therefore complex since the term “disability” encompasses a range of meanings and conceptualizations.

For the purpose of this article, and in line with the American Psychiatric Association (2022), we refer to IDD as cognitive and adaptive functioning impairments, meaning challenges with everyday living skills. Youth with an IDD may have difficulties in problem-solving, concentrating, and learning new things. IDDs may have a wide array of origins or causes and may include many different diagnoses. Cognitive disabilities, organic brain injury (e.g., FASD), traumatic brain injury (e.g., an accident in childhood) are a few examples. Intelligence or IQ is an important criterion in an intellectual disability. A young person can have a developmental disability *without* an intellectual disability (e.g., high-functioning autism spectrum disorder or cerebral palsy). IDD is characterized by adaptive functioning based on the following primary areas: conceptual, social, and practical. Likewise, we recognize that young people with IDD often experience multiple social disadvantages that contribute to justice involvement, including social and economic issues, early life experiences, and previous justice involvement. According to Kincaid (2017), a lack of support and services coupled with involvement in the justice system may further complicate life issues for youth with IDD. We also recognize that disabilities are intricately tied to the Legacy^1^ (Bendo et al., 2021; Benn-John, 2019; Johnstone and Lee, 2024) that have devastated Indigenous communities across Canada (Dowse et al., 2014).

While some literature has focused on “neurodevelopmental disorders” in a youth justice context, most of this research has been conducted in New Zealand, the United States, and Britain. Even within these contexts, scholars have noted the lack of research focusing on neurodevelopmental disorders, and the underlying causes of over-representation in the justice system (see Kincaid and Sullivan, 2020). This scholarship focuses primarily on assessing how youth with disabilities are treated, and what improvements can be made to the system. Our article differs in that it focuses specifically on the justice conceptions and experiences of crown attorney and defense counsel working with youth with IDD in Ontario, Canada.

### Justice, Situated Judgement, and Youth with Intellectual and Developmental Disabilities

The function of lawyers and legal professionals has considerably changed since the early days of the profession in the nineteenth century. The broad scholarship on lawyers, law firms, and the legal profession in general reveals this transformation. This literature demonstrates that the work of lawyers and the organization of the profession in firms and corporate offices are molded by manifold interconnecting factors, such as emotional labor, socio-cultural dynamics, market competition, organizational forms, institutional change, and conflict over jurisdiction (Cooper et al., 1996; Faulconbridge and Muzio, 2008; Leclerc et al., 2020; Muzio et al., 2013; Reich, 2020; Wallace, 1995, 1997).

At the institutional level, North American lawyers are shaped by what Kagan (1994: 2) refers to as “adversarial legalism.” He avers that while adversarial legalism “stems primarily from enduring features of American political culture and governmental structure, the legal profession itself does play a significant independent role in promoting and perpetuating adversarial legal contestation as a prominent feature of governance.” This system is a collection of “legal propensities” for the purpose of policy making and dispute resolution. These tendencies are categorized by “high degrees of formal legal contestation, litigant activism, and substantive uncertainty” (Kagan, 1994: 4). Adversarial legalism is also described as being firmly hierarchical, as it is grounded in an “authoritative legal official or body” governing the procedure and principles of legal practice (Kagan, 1994: 4).

This system of adversarial legalism can create psychological tension for lawyers, who are tasked with upholding professional comportment, like detachment, in the face of hostile and adversarial environments. For Yakren (2008: 154), the focus on professional detachment positions lawyers as “mere agents” when in fact they may feel “more like culpable principals.” Detachment requires an unrealistic standard for lawyers, leading to psychological stress from being incapable of meeting “professional” demands that lawyers feel are required to perform their work (Yakren, 2008: 154). A significant moral issue, some lawyers are in persistent conflict with their professional selves as they wrestle with moral afflictions when representing clients (Yakren, 2008: 157). Yakren (2008: 168) avers that no matter how inflexible professional rules about ardor and disinterest are, there is inexorably going to be circumstances where a lawyer's “experienced emotions do not mesh with the prescribed role,” which has been observed to lead to experiences of emotional dissonance.

Concomitantly, legal professionals also have their own conceptions of justice that may or may not be met by the courts. These often cohere and depart from popular and academic conceptions of justice. Inspired largely by the work of Lind and Tyler (1988) on procedural justice in the 1980s, researchers have examined perceptions of fairness in the conventional criminal justice system. This focuses on two forms of justice: distributive and procedural justice. Distributive justice refers to people's moral evaluation and actions in response to the allocation of rewards and punishment. It is promoted when outcomes are consistent with certain implicit norms for the allocation and distribution of resources, such as equality of outcome or need. Procedural justice is assessed in terms of people's perceptions of being treated procedurally fair; this can be assessed in terms of a person's engagement with police and/or the courts. Procedural justice is dependent on being treated fairly and having a voice in matters of concern, and upon the perception that decision makers were neutral and transparent and expressed trustworthy motives (Lind and Tyler, 1988; Tyler and Lind, 2002). Such just ends are subject to broader contextual factors with which lawyers must contend, and conceptions of the just are variable and do not automatically conform to adversarial ends of winning a trial, that is, achieving distributive justice. Whereas procedural justice conveys a sense of fairness in treatment, its customary application does not offer a way of assessing manifold practices of courts and work in legal settings. Indeed, lawyers may have their own conceptions of justice for clients, victims, and society more generally that operate as a criticism of the criminal justice system, as well as an assessment of the material and temporal constraints that legal professionals may face with defending and prosecuting specific offenders and offenses. Such an understanding of justice and criticism of the system itself points to the situated moral expectations that are specific to the context under which legal professionals work.

To apprehend both the ways in which legal professionals comprehend justice and the context in which these comprehensions are situated, we turn to the work of Luc Boltanski and Laurent Thevenot's (2000) sociology of situated judgment. Boltanski and Thevenot (2000) propound a model for comprehending the everyday sense of justice and moral expectation articulated in ordinary situated disputes. This model is founded upon their work on justification (Boltanski and Thevenot, 2006), which examines people's moral evaluations in everyday situations, and the way in which moral justifications are evaluated based on repertoires of justice and notions of the common good. Justifications refer to the act of providing reasons for the validity, legitimacy, and defensibility of an action, belief, or social arrangement (see Susen, 2017). Legitimate justifications of what is morally acceptable follow recognizable patterns of moral orders and moral cognition—orders of worth—that permeate social situations. These orders of worth represent social conventions on which people rely to justify their actions, make ethical claims, or resolve contentions situations in the name of justice (Levi et al., 2022). This approach explicates how people determine what common goods are worth pursuing, and the appropriate means or economies—rational arrangements, material resources, effort, and so on—that contribute to the pursuit of the common good (Grattarola, 2023). Justifications provide a moral grammar that reflects “collectively held ways of evaluating and determining moral worth in social life—for conceiving of one's own worth, for evaluating the worth of others, and for articulating standards by which to engage in critique and to resolve disputes” (Levi et al., 2022: 90).

Boltanski and Thevenot (2006) identify six orders of worth—market, industrial, civic, domestic, inspired, and fame each with its own logic and value system. Scholars have employed the analytical framework of orders of worth to identify the moral grammars invoked in contentious situations. For example, in Levi et al.'s (2022) study of interactions between police and arrested persons, individuals utilize two orders of worth that position the police and themselves in relation to one another: a civic order of worth, through which claims of fairness, polity, and legal rights and rules are articulated, and a domestic order of worth through which statements about community, empathy, local knowledge, and personal experience are articulated. Overall, the orders of worth approach posit that people possess and exercise natural critical competence that enables them to formulate beliefs about the appropriate ends and the effective means to achieve those ends collectively (Grattarola, 2023). These beliefs are subject to justification through their articulation and contestation in intersubjective, in situ exchanges that bring together the moral cognition of actors with the practical considerations and constraints of collective action. Specific social arrangements that come under critical scrutiny are subject to “test of worth”; that is, argumentative frameworks that provide the foundation for evaluating the strength of public arguments derived from particular orders of worth (Boltanski and Thevenot, 2006). This approach underscores the role played by actors seeking compromise when certain institutional arrangements are in dispute and is, therefore, well suited for analysing criticisms of justice system arrangements and disagreements about the legitimacy of justice processes, procedural fairness, and the rights of young people (see Patriotta et al., 2011).

In this context of justice, orders of worth are tools to explain an everyday sense of principles of justice, providing a normative everyday orientation that “performs, as a reflexive turn on the sense of justice, an operation of rule-setting, of determination of different types of common good, and of legitimate or illegitimate relations between these common goods.” (Boltanski and Thevenot, 2000: 210). Susen (2017) expounds upon the essential role of justifications for the principles of justice in shaping society to coincide with the common good. The situated nature of justice means that the common worlds in which we exercise justice are regulated by different “principles of justice” that reflect people's relative moral values and orders of worth. The concept of a common good is therefore contingent upon the social setting in which it emerges, where people produce and reproduce morally codified worlds. The principles of justice that undergird specific settings are therefore negotiated constantly by those within them. Negotiated principles of justice are rarely neutral, given the heterogeneity of resources people are able to mobilize in defense of their moral positions or enact their versions of justice. Justifications for justice are always laden with contextual factors, values, perspectives, interests, and tensions.

In this article, our focus is on normative issues involving civic and market justifications situated in a criminal justice context. We rely on this framework due to the fact that such an approach goes beyond conventional notions of justice—distributive and procedural justice—and considers how varying spheres of justice commingle within spaces of contestation like courts. Such perspective reveals how people contest perceived injustice in a plethora of ways under conditions of neoliberal capitalism (cf. Boltanski and Chiapello, 2005). In addition to these previously identified forms of justification, we offer an additional justification that is possessed of broad application but also specific to the context of youth criminal justice institutions—a *disability justification*. The disability justification represents an order of worth where legal professionals rely on conceptions of disability to criticize ableist processes and procedures in the court, such as presumptions about an able accused who is capable of understanding the charges against them or the processes to which they are submitting. The disability justification constitutes a moral grammar critical of courts for lacking the capacity to support young people with intellectual and developmental disabilities and, principally, for lacking an orientation to, and understanding of, young people with IDD. This justification coheres with broader orders of worth in relation to disability that operate outside of youth courts. Such justifications involve challenging the physical composition of spaces, the way in which technologies are created with “normal” conceptions of being, and how the semiotic terrain applies language that is exclusionary of those with manifold disabilities.

## Methods

This article draws from semi-structured interviews with legal professionals in Ontario, the most populace province in Canada, who were employed in the area of youth criminal justice. Non-probability sampling, particularly purposeful and snowball sampling, were employed to recruit crown attorney's (prosecutors) and defense counsel. Through this sampling method, participants were selected based on similar characteristics: their profession and focus on justice-involved youth with IDD. The semi-structured interviews with legal professionals were conducted from May 2021 to April 2022. The sample was specific to those whose work was directed by the YCJA in order to gain valuable insights into the project's main objective. The interviews ranged in length from 60 to 90 min, while some interviews exceeded this timeframe. Whereas the daily lives of citizens across the world were significantly altered by governments attempts to limit physical interaction and reduce contagion, qualitative researchers turned to remote and innovative approaches to conduct research during the COVID-19 global pandemic, be it on the experiences of the pandemic or other areas of research (Pocock et al., 2021; Rahman et al., 2021; Vindrola-Padros et al., 2020). We followed suit as interviews were conducted during the COVID-19 global pandemic and were facilitated through the online platform *Zoom*. We experienced difficulties contacting and recruiting from some regions of Ontario, but Zoom interviews were cost and time-efficient, flexible in terms of scheduling, and allowed us to overcome geographical distances that were constrained due to pandemic restrictions. Similar to other qualitative research projects using Zoom (Oliffe et al., 2021), despite the loss of in-person interview nuances of virtual interviews, we were able to comfortably interview participants from home or their offices and provide rich therapeutic value to legal professionals about their experiences of and struggles working within the youth legal system with young people with IDD.

The interviewers utilized a semi-structured interview guide, asking participants open-ended questions regarding their experiences of working within the youth criminal justice system. Researchers obtained multi-dimensional responses as the interview was divided into specific themes relevant to the study objective, ranging from participants’ professional background to applications of the YCJA, knowledge about and experiences with IDD, justice system support, and youth rights. Moreover, in utilizing a semi-structured interview guide, interviewers are able to capture participants’ lived experiences as well as the theoretical nature of the phenomenon under study (Galletta and Cross, 2013). All interviews were audio recorded and transcribed by the research team. Names and personal information were omitted, and pseudonyms were used during transcription.

Transcripts were analyzed according to thematic derivation procedures: description and analysis, as outlined by Wolcott (1994). The description phase involved defining and describing justice professionals’ viewpoints on working in the youth criminal justice system. A phenomenological approach to analysis was favored as it supported our aim to analyze how legal professionals interpret and engage with their experiences working with justice-involved youth with IDD (Freeman, 2011; Schwandt, 1999). This approach is useful as it enables researchers to gain an expressive viewpoint of the participants’ experiences and an opportunity to interpret participants’ insights at a structural level (Creswell, 2012). Analysis involved a deeper exploration of the data as the researchers identified common patterns in the data and analyzed similarities to produce themes. Color coding techniques were used to identify patterns and turned into themes based on recurring commonalities. In identifying patterns within the data, an in-depth narrative of the phenomenon was produced through the thematic statements (Lochmiller, 2021). Multiple research assistants completed this process independently and engaged in discussion to assess the validity of the themes generated. This procedure led to an understanding of justice professionals’ viewpoints on conceptualizing justice in relation to young people with IDD, in the absence of formal guidelines regarding justice outcomes for youth. In adopting Boltanski and Thevenot's sociology of situated judgment as an approach to analyze legal counsel's interactions with young people with IDD who are in conflict with the law, we examine the themes related to legal professionals’ conceptions of justice.

## Results

In keeping with scholarly work utilizing Boltanski and Thevenot (2000), we identify “economies of worth” on which people rely to make ethical assertions to resolve antagonistic situations in the name of justice (Levi et al., 2022; Susen, 2017), and which inform our analytical framework. In relation to our present work, these include claims oriented to *market justifications* regarding costs of providing services to young people with intellectual and development disabilities, civic justifications focusing on formal legal rights and equality, and disability justifications that criticize formal legal processes and procedures that fail to accommodate young people with IDD. These we elucidate below.

### Market Justifications

In the following, we consider justice professionals’ market justifications situated within a youth justice context. Market justifications focus on competitiveness, value, and the possession of rare goods and services. In the context of youth justice, lawyers framed their claims to worth in a market for scarce resources and services and young people's ability to access those services. Within the context of austerity, this was typically framed in terms of funding, cost, affordability, resourcing, and intervention. Evidence from the interviews shows a deep concern for affordability of services, access to services through the criminal justice system, the cost of early diagnosis and intervention, and a broad concern for thinly supported markets offering crucial interventions and diagnostic services prior to a young person's justice involvement. More precisely, justice professionals framed their concerns about justice in terms of the affordability of services for young people, inequalities in the pathways used to access those services, and, broadly, the absence of funding for community services to support those with IDD.

The most significant gap—the affordability of services—was characterized by the high cost of diagnostic and treatment services for youth with IDD, creating a substantial barrier for families and young people who are rarely able to afford these services. Participants believed that lack of access to services, especially early intervention and treatment, were responsible for a young person's justice involvement. Several justice professionals observed:

**Participant #22:** Treatment can be very expensive for most families. If it involves, for instance, hiring a psychologist, or a social worker, or somebody who is not covered by OHIP, then that is a cost that will be born, usually, by the family, sometimes that will be covered perhaps by insurance programs that one of both parents have, but not always, and not in an unlimited way.

More salient is the observations that many of the families struggling with justice-involved young people can hardly afford the cost of diagnostic and treatment services for their children. The following excerpts echo these sentiments:

**Participant #36:** Keeping in mind that a lot of a lot of youth with IDD come from backgrounds that may have not allowed them to see doctors or to engage in treatment or to be diagnosed or to be accommodated anyway.

**Participant #1:** A lot of the families that I deal with who get involved in criminal justice system don't have extra money to get diagnoses. A—they don't have the money to. B—they don’t even know they have to pay for it. So, there's a lot… I mean it's like an onion. First of all, they might not recognize there is an issue. Second of all, they might not know where to get help. They might not know whether they have to pay to get a diagnosis or help. And it's really sad that it's always the, the people who are less off, you know, get kind of left behind.

In the context of this study, market economies of worth frame injustice through the absence of affordable support for families and young people, an absence that many professionals see as responsible for their client's criminal involvement. When discussing the absence of funding for services to support young people, lawyers offer a market justification focused on the need for a general level of social and economic support for youth with IDD. This need was often expressed through a call for more overall funding for community and educational support, as expressed by a Crown attorney:

**Participant #12:** It's hard as a crown attorney. You’re not supposed to say too much. I wish there was more funding to keep young people in school. I wish there was more funding to help young people find meaningful work. I wish there was, in terms of intellectual disability, more being done to not stigmatize young people when they're seeking out help.

Participants believed that the YCJA has been successful in advancing justice for young people, particularly through a reduction in prosecutions and custody, the use of community-based programs, and through the promotion of rehabilitation. However, against the backdrop of these successes, they offer a market justification that evinces that justice for young people is limited by a lack of resources and funding:

**Participant #13:** I don't think the YCJ could be more committed to identifying and promoting rehabilitation for these kids who have these challenges, but I think that the administration, like the resources and just the ideas that we're using to administer those resources could be improved.

Echoing this market justification, one respondent from a major metropolitan center notes:

**Participant #10:** I have been told sometimes by youth workers that they feel there's a limit on some of the resources or maybe the travel requirements for some families to access a good clinic or a good option are simply too great to make it realistic, but that's not a problem with the statute, that's really more a problem with both federal and provincial governments … ensuring that adequate resources are behind the statute, so that those of us on the front lines can adequately take advantage of what it offers.

In accordance with neoliberal rationalities of framing all spheres to economic terms and metrics, concerns of youth justice cede to the mandates of economic austerity (Brown, 2015). As such, the absence of funding was a consistent lament across nearly all respondents, which they linked directly to a myriad of concerns: undiagnosed IDD, FASD, cognitive, and learning disabilities; frustration among parents and professionals; and long wait lists for services. For most, the correlation is clear: there would be many fewer young people in the criminal justice system if adequate support existed to flag, diagnose, and treat them for IDD and other issues before they became contributing factors to criminality.

### Civic Justifications

In this section, we analyze justice professionals’ civic justifications for their everyday evaluations working with youth with IDD in the justice system. These justifications shed light on how the participants comprehend justice (in relation to youth with IDD) as well as the contexts that shape these comprehensions. In conceptualizing civic justifications/civic orders of worth, there is a primary focus on collective welfare, equality, and solidarity. In this context, worth is attained by sacrificing individual interests and focusing on collective interests (i.e., solidarity with others). This is evidenced in the demand for formality, procedures, rules, regulations, legal frameworks, fundamental rights, welfare policies, formal legal entitlements, all of which define what is best for the common good. As a result, the insights and arguments expressed by our participants focus specifically on procedural fairness for young people, youth rights, an acknowledgment of barriers/disadvantages youth experience as they try to navigate the complex justice system, and the impact of these systemic barriers on young people's experiences/outcomes. As evidenced below, these civic justifications have a strong relationship to disability justifications which we unpack further in the next section.

The first civic order of worth that we analyze focuses on the participants’ justifications around the silencing of youth as they move through the youth justice system. The participants emphasize that the youth justice system is not set up to listen to the viewpoints or perspectives of the accused. Rather, the system is designed to be intimidating, it is inaccessible to most social groups, it is formal, strict, and is not designed in a youth-friendly way that enables or encourages youth participation. Within the system are the actors that operationalize systemic rules and policies. The actors involved in the system (i.e., judges, lawyers, etc) will have an impact on the youth that they serve (i.e., how they treat youth and how they interact with them). As the participants point out, this is context-specific, and each justice professional will vary in their approach to youth engagement. These civic justifications highlight that it is difficult for youth to feel heard throughout the youth justice process, especially in intimidating contexts, such as courtrooms, given that these processes were not designed to amplify the viewpoints of the accused. Whereas this may be difficult for neurotypical youth, those with IDDs, who may not receive individualized support, may find it particularly difficult to understand and navigate the justice process. Consider the following interview excerpts:

**Participant #10:** I imagine many youth will feel no one listens to me, no one's even asked me what I want to say about this or what I want to do. Unless you have an excellent lawyer who's constantly calling you and talking to you and saying, how do you feel about this, what do you want me to tell the judge or the crown attorney.

**Participant #19:** It's very hard in the criminal justice system for anybody who's in the accused position to feel heard. Generally, you're on the pathway to “how do I get, you know, outta here. I just want to get out of this place, I don't want anything to do with these people”. It's very formal, you’re all dressed in suits and ties and it's a very formal proceeding. Right, your judge's sitting on a podium, you're addressing somebody who's got a sash on them, there's not… This isn't the soft room, right. I don't know how much somebody who already suffers from a disability and probably suffers from significant stigma in society as they arrive, they're already suffering from that, probably having a stigmatism in school, to say that they're going to be heard and they have an opportunity to be heard. Will they be asked if they want to say something? Will they be asked questions? What an intimidating environment for a young person, we're talking about people, they have diminished moral blameworthiness, and yet you're asking the same person with diminished moral blameworthiness to process and respond in a courtroom. Wow, that's difficult.

**Participant #24:** Every client is very different and has individual needs, and for someone that has any kind of disability, then I have to go about their file differently and advocate in different ways and make sure that the crown and the system is aware of the situation and understands, you know, what they're going through and what they're dealing with because then they will be dealt with differently.

Participants 10 and 19 reveal that there is a shared understanding amongst the justice professionals regarding the social exclusion of youth in justice processes. As participants 10 and 24 highlight, the justifications for navigating this issue come down to the role of counsel and if they work closely with youth with IDD to help them understand the process, how they explain the process to them, the strength of their relationships with their clients, as well as the consistency of these relationships. The participants’ civic justifications are showcased by a sense of solidarity with young people with IDD who are involved in the justice system given the systemic barriers they face, inequity, silencing, and social exclusion. In addition to broader civic justifications that shed light on the silencing of youth, there are intersections with disability justifications that participants allude to above. For instance, they outline how difficult it is to navigate the justice system in general especially when there may be an IDD that impacts a young person's ability to comprehend/process information. This is again where lawyers play a critical role in advocating for their clients, supporting their youth clients, and explaining to them what is happening and how they can proceed. These civic justifications work in relation to disability justifications as we will unpack further in the next section.

### Disability Justifications

Another key justification that emerged in our interviews with participants is the moral arguments that justice professionals identify surrounding disability in legal contexts. The disability justification therefore represents an order of worth that the participants engage to critique ableist processes and procedures that impact youth with IDD. Disability justifications include a recognition of the barriers youth with IDD experience, moral arguments about how to support youth with IDD in the justice system, as well as complexities around comprehension and adherence to legal processes and outcomes. The following excerpts showcase the various disability justifications that legal professionals use to describe their notions of justice in the absence of formal guidelines that accommodate the needs of young people with IDD. Further, they outline various justifications that describe the complicated conditions that they work under in the justice system to support young people with IDD. We first present excerpts relating to the struggles youth with IDD experience in the justice system:

**Participant #19:** I think they struggle in the criminal justice system like any other avenue in life. I don't think the criminal justice system suddenly makes it easier for people with IDD to navigate the ins and outs of it - there's so many rights and issues that you have to understand to get through the criminal justice system that processing it, understanding it, actively participating in your own, what's your own outcome, is more difficult if you have an IDD, and everybody has to work hard to make sure that they take it into it account.

**Participant #7:** It's their first time because it's an unfamiliar process and I think that's amplified when, [it] is a young person who doesn't have life experience and then further amplified if they have IDDs which can make it a little more challenging of a process for them.

**Participant #11:** The average person, in air quotes here, has enough trouble understanding it, let alone a young person, let alone a young person who has any kind of need, whether it's related to mental health or developmental disorders.

These quotes reflect a disability order of worth, through which the justice professionals articulate claims of worth. These claims focus on the struggles youth with IDD experience in the justice system. For instance, as Participant 19 outlines, struggles surrounding the processing of information, understanding the various stages and procedures of the justice system, and ultimately, participating and engaging with the system. Participant 7 reinforces how navigating the system is difficult for any young person, particularly a youth with IDD who may not have the support required to understand the various stages. Participant 11 echoes sentiments from Participants 19 and 7, explaining that there are complicated procedures and processes involved in the justice system, and these are amplified for youth who may have an IDD. As a disability justification, participants evince that the justice system is not designed in an accessible way that enables youth to understand the various processes that they are required to engage in. Rather, the system is built on universalized, adult-centric, ableist frameworks that oftentimes silence, govern, and control youth. In an attempt to navigate these issues, the participants highlight how worth is attained by sacrificing individual interests and focusing on collective interests (i.e.,: solidarity with youth by providing enhanced support). In what follows we provide excerpts that reveal the way in which participants offer a disability justification that indicts the broader youth justice system that contributes to the poor outcomes for young people with IDD:

**Participant #3:** I think certainly there are cases where there are gaps. And a lot of it is, is, again, I hate to say this, is really dependent on the Crown, the defense, the court … if they don't have information or an appreciation of that particular disability, then they can't be treating the child properly, right?

**Participant #33:** What the justice system expects of that particular youth will have to be different than what it expects of someone who is neuro-typical, for example. I think that defence counsel does relay those concerns to a judge whether it's on sentencing and making sentencing submissions and what the appropriate sentence should be. I think the crown will also take it into account when they make their decisions about what sentence for disposition should be given to a young person, they have to take into account that particular person's level of functioning, for example, their ability to take responsibility for something may depend on their level of understanding.

In relation to participants 3 and 33, they indicate that the gaps within the system and individual actors as leading to injustices. This disability justification involves the lack of information, specifically in relation to the specific disabilities of the young person in court. Such criticism also reveals the perceived shortcoming of the courts in understanding the diminished moral blameworthiness of the young person. Such a qualification stands against the stated orientation and framing of young people within the Canadian Youth Criminal Justice Act. The next set of quotes therefore speak to the disability justifications that legal professionals outline in relation to how they support youth with IDD and the conditions they work under as a result of the justice system's flaws:

**Participant #1:** I think if you have a client who was diagnosed with an intellectual development, you have to, from a moral standpoint, advocate for and try to explain why that might have caused, you know, criminal behavior. Or lack of empathy towards certain acts, and to also explain to your client and have them appreciate why what will happen was not right. Overall, it is taking more time to it.

**Participant #7:** People often try to baby people with IDDs and sort of, talk to them in a childish way which I don't like to do. It's important to provide individual service in which they can relate, and they could understand, so I try not to do it too much but if it's required and if that's what that individual needs, then I think it's important for you to provide your service in a way that they could understand it.

**Participant #30:** You know, I wouldn't talk to them the same way that I would speak to somebody else. If I knew that there was a cognitive delay, I would make extra efforts to spend time to make sure they understood what was happening because I would appreciate that. Probably that would be more difficult for them. I would try for the same outcomes but try to offer more support, of course.

As disability justifications, participants personalize their interventions into matters related to working with youth with IDD. In relation to participant #1, the defense counsel engages in an individual response to systemic issues related to the disabilities of his clients, while articulating the implications of criminal behavior to his clients in a “patient” way. Similarly, participants #7 and #30 maintain a disposition towards youth with IDD that involves a recognition of their disability but do not devolve into an infantilization of young people. It is gauged in accordance with the individual needs of the young person. Disability justifications here and above serve as a broader critique of the youth justice system. Accommodations for young people with IDD are viewed as a common good, but as the legal professional's justifications demonstrate, the lack of resources, competencies of court formalities, and legal lexicon disenfranchises youth from meaningful participation in the youth justice system.

## Discussion and Conclusion

Relying on Boltanski and Thevenot's (2000) model of situated moral judgment, we analyzed legal professionals’ reflections on and criticisms of their work environments in relation to the positioning of young people with IDD who are in conflict with the law. We examined the language they employed to express their criticisms of legal situations presented throughout the youth justice system process, the material conditions under which they work and the constraints to adequately provide for youth with IDD, and their conceptions of justice in the absence of prescribed guidelines regarding how to accommodate the needs of young people and ensure the protection of their rights. Our analysis shows that legal professionals engage in three orders of worth that correspond to market, civic, and disability justifications that are the basis of evaluation and criticisms of the youth criminal justice system and its treatment of young people with IDD.

The moral justification approach deployed here goes beyond and contributes to conventional approaches to justice and criticism in a number of ways. First, this article apprehends, under neoliberal austerity policies, the material constraints that justice professionals identify and how those funding supports mean that youth with IDD are underdiagnosed and are not provided much-needed services. Second, through exploration of civic justifications, we contribute to work on procedural fairness and youth justice, by exploring how justice professionals criticize the impediments and difficulties youth with IDD experience as they make their way through the youth justice system and the implications of this system for justice outcomes and their continued involvement in this system. Such an antagonistic circumstance invariably means that young people's voices are silenced, which is further complicated when the young person has an intellectual and developmental disability. Further, by developing the concept of disability justification, we attend to the orders of worth regarding perceived shortcomings concerning accommodations and services available for youth with IDD. In addition, our analysis of legal professionals’ responses offers insights into the ways in which our participants attempt to enframe the source of the insufficiencies regarding disabilities in the youth court system.

Our findings resonate with research outside of Ontario, Canada to broader international contexts. In Australia, the US, and the UK, young people with IDD face difficulties at all levels of the criminal justice system (Chester, 2018; Dowse et al., 2014; Hayes, 2007) in similar ways outlined in this article. That said, our mobilization of Boltanski and Thevenot's model of situated moral judgment offers a basis by which to analyze how those working within criminal justice systems pose criticisms of these systems and attribute worth to specific forms and avenues for justice. Our article demonstrates the value of utilizing this model and attendant concepts to examine the manifold ways professionals working in justice contexts criticize the conditions under which they work and pursue justice. In relation to youth with IDD, our concept of disability justification, within this model, offers a fruitful way of analyzing disability and accommodation within institutions.

## Notes

1. The Legacy, here, refers to the Canadian government's systematic efforts to “civilize” the Indigenous population through sending 150,000 Indian children to residential schools between 1867 and 2000 and erase this population through the forced adoption of 20,000 Indigenous children between 1950 and 1985.
